# Adenosine A_2A_ receptors control generalization of contextual fear in rats

**DOI:** 10.1038/s41398-023-02613-0

**Published:** 2023-10-12

**Authors:** Ana P. Simões, Marina A. M. Portes, Cátia R. Lopes, Felipe Vanz, Vanessa S. Lourenço, Anna Pliássova, Ingride L. Gaspar, Henrique B. Silva, Ângelo R. Tomé, Paula M. Canas, Rui D. Prediger, Rodrigo A. Cunha

**Affiliations:** 1 CNC- Center for Neuroscience and Cell Biology, University of Coimbra, 3004-517 Coimbra, Portugal; 2 Department of Pharmacology, Graduate Program in Pharmacology, Center of Biological Sciences, Federal University of Santa Catarina (UFSC), Florianópolis, SC Brazil; 3 Faculty of Science and Technology, Department of Life Sciences, University of Coimbra, Coimbra, Portugal; 4 Faculty of Medicine, University of Coimbra, 3004-504 Coimbra, Portugal; 5 Multidisciplinary Institute of Aging (MIA-Portugal), University of Coimbra, 3004-504 Coimbra, Portugal

**Keywords:** Long-term memory, Molecular neuroscience

## Abstract

Fear learning is essential to survival, but traumatic events may lead to abnormal fear consolidation and overgeneralization, triggering fear responses in safe environments, as occurs in post-traumatic stress disorder (PTSD). Adenosine A_2A_ receptors (A_2A_R) control emotional memory and fear conditioning, but it is not known if they affect the consolidation and generalization of fear, which was now investigated. We now report that A_2A_R blockade through systemic administration of the A_2A_R antagonist SCH58261 immediately after contextual fear conditioning (within the consolidation window), accelerated fear generalization. Conversely, A_2A_R activation with CGS21680 decreased fear generalization. Ex vivo electrophysiological recordings of field excitatory post-synaptic potentials (fEPSPs) in CA3-CA1 synapses and of population spikes in the lateral amygdala (LA), showed that the effect of SCH58261 is associated with a reversion of fear conditioning-induced decrease of long-term potentiation (LTP) in the dorsal hippocampus (DH) and with increased amplitude of LA LTP in conditioned animals. These data suggest that A_2A_R are engaged during contextual fear consolidation, controlling long-term potentiation mechanisms in both DH and LA during fear consolidation, impacting on fear generalization; this supports targeting A_2A_R during fear consolidation to control aberrant fear processing in PTSD and other fear-related disorders.

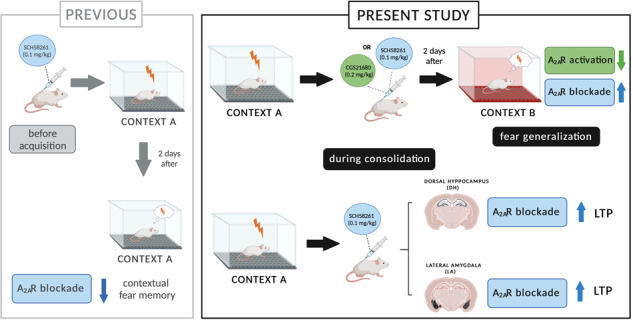

## Introduction

Anxiety, stress and post-traumatic stress disorders (PTSD) involve an abnormal fear response and overgeneralization [1]. The study of adaptive and maladaptive processing of aversive memories, including fear generalization, has largely used rodent models of fear conditioning since the neural circuitry that encodes associative fear memory is conserved across mammals [2]. This has revealed that fear conditioning and fear generalization critically involves the hippocampus and amygdala, among other brain regions [3, 4]. The hippocampus processes contextual cues [5, 6] and promotes the association between context and fear through direct and indirect projections to the amygdala [7]. The amygdala allows associating sensory cues with aversive stimuli [3]. Contextual fear learning and consolidation in particular, is thought to rely on synaptic plasticity mechanisms in both hippocampus and amygdala [3, 4, 8]. Abnormal consolidation of fear memories is proposed to explain fear overgeneralization but the underlying mechanisms are not completely understood [9]. Likewise, clinically safe and efficacious pharmacological interventions to interfere with fear overgeneralization still need to be developed.

Adenosine A_2A_ receptors (A_2A_R) modulate plasticity processes in many brain regions, namely in hippocampus, impacting on memory function [10]. Moreover, A_2A_R emerges as a promising target to regulate mood and memory since repeated stress triggers an upregulation of hippocampal A_2A_R [11] and A_2A_R antagonists limit or counteract memory and mood changes in chronically-stressed rodents [11–13]. Additionally, triggering A_2A_R-mediated signaling in the hippocampus is sufficient to cause memory impairment [14–16]. However, limited information is available on the role of A_2A_R in fear conditioning and especially in the different phases of fear memory processing. It was previously shown that alterations of the extracellular adenosine levels modify cued fear conditioning [17]. Furthermore, forebrain A_2A_R are essential for normal fear acquisition and retrieval and deletion of hippocampal A_2A_R alone impairs contextual fear memory [18]. In addition, A_2A_R in basolateral amygdala control long-term potentiation (LTP) and blockade or downregulation of A_2A_R in this brain region disrupts learning of associative fear [19]. Corroborating animal studies, it was found that polymorphisms of the A_2A_R gene are associated with anxiety and panic disorders in humans [20].

Since it is currently unknown if A_2A_R can control fear consolidation and generalization, we now tested the impact of systemic administration of selective antagonist and agonist of A_2A_R within the consolidation time-window of context-associated fear memory and probed the consequences for fear generalization in rats.

## Material and methods

### Animals

The experiments were carried out in male adult Wistar rats (*Rattus novergicus*) between 12 and 16 weeks of age and weighing between 270 and 350 g. The animals were kept grouped in cages, with a maximum number of 5 animals per cage under a light/dark light cycle of 12 h, constant temperature of 22 ± 1 °C, with free access to water and food. All behavioral tests were performed during the light cycle. The study was performed in accordance with the principles and procedures outlined as “3Rs” in the guidelines of the European Union (2010/63/EU), FELASA and ARRIVE and was approved by the Ethics Commission on the Use of Animals of the Federal University of Santa Catarina (protocol no. 5218190418) and by the Ethics Committee of the Center for Neuroscience and Cell Biology of the University of Coimbra (ORBEA 238-2019/14102019).

### Drugs

7-(2-phenylethyl)-5-amino-2-(2-furyl)-pyrazolo-[4,3-e]-1,2,4-triazolo[1,5-c]pyrimidine (SCH58261; a selective A_2A_R antagonist; Tocris, USA) and 2-p-(2-carboxyethyl)phenethylamino-5′-N-ethylcarboxamidoadenosine (CGS21680; a selective A_2A_R agonist; Tocris, USA) were dissolved in saline containing 10% dimethylsulfoxide (DMSO) and administered systemically by intraperitoneal (i.p.) injection at the doses of 0.1 and 0.2 mg/kg, respectively (in a volume of 1 mL/kg) immediately after the fear conditioning session, unless otherwise specified. The doses and concentrations used in this study were chosen based on previous studies (see [11, 15, 21]). For the electrophysiological experiments testing the modification of adenosine A_1_ receptor (A_1_R) function, we used the closest but stable chemical analog of adenosine, 2-chloroadenosine (CADO, from Sigma-Aldrich, Portugal) in a previously validated concentration range of 0.1–1 μM [22] as well as the selective A_1_R antagonist, 1,3-dipropylcyclopentlxanthine (DPCPX, from Tocris), used in a supra-maximal and selective concentration of 100 nM [23]. It should be noted that although CADO can activate A_1_R and A_2A_R, the fact that A_2A_R are devoid of effects in the control of basal synaptic transmission [24, 25], allows using CADO to selectively probe the efficiency of A_1_R to control hippocampal synaptic transmission [22, 23].

### Contextual fear conditioning

For the contextual fear conditioning, the animals were exposed individually in a rectangular box (35 × 20 × 0 cm) with aluminum side walls, front wall and acrylic ceiling, and gridded floor with stainless steel bars of 3 mm in diameter, spaced by 9 mm (Insight, Ribeirão Preto, Brazil). During a first exposure (except for Supplementary Fig. 1G), called the familiarization session or habituation, the animals freely explored the box for a period of 3 min, without the presentation of any aversive stimulus. On the next day, the animals were re-exposed in this same box for the conditioning session (or pairing), during which the formation of associative aversive memory was induced. During this session, after an initial period of 30 s (pre-shock period, except for Supplementary Fig. 1G), an electric shock (with different intensities specified for each experiment) was applied to the paws of the animals (lasting 3 s) through the gridded metal floor attached to an electric current generator. The conditioning session was classified according to the intensity and number of electrical stimuli, as follows: i) weak, with the presentation of 1 shock of 0.5 mA; ii) intermediate, with the presentation of 3 shocks of 0.7 mA and; iii) strong, with the presentation of 3 shocks of 1.2 mA [26, 27]. The interval between the shocks in the protocols of intermediate and strong intensity, was 30 s. After the conditioning session, each animal remained in the box for an additional 30 s (post-shock period) before returning to its home cage. Pharmacological manipulations occurred immediately after the conditioning session to modulate the initial stage of contextual fear memory consolidation, or in the particular case of the experiments summarized in Fig. 2A, at 3 or 6 h after the conditioning session in order to modulate later phases of contextual fear memory consolidation.

**Fig. 1:**
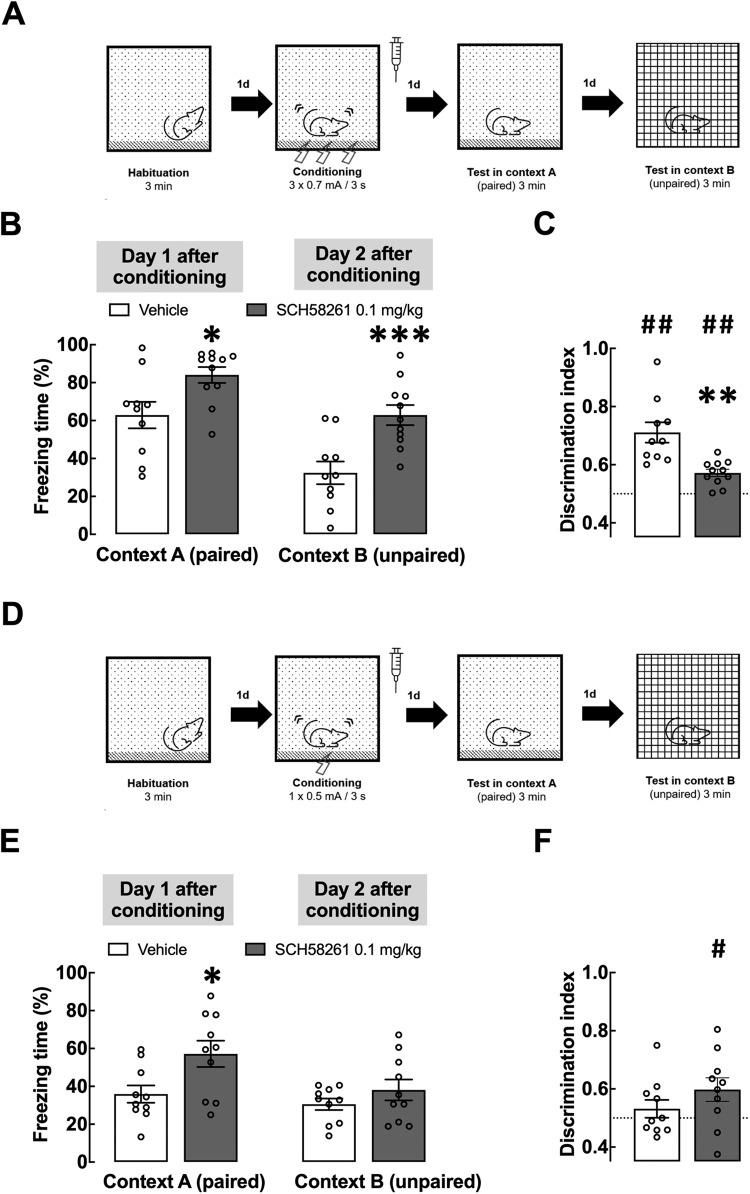
Blockade of A_2A_R immediately after contextual fear conditioning increases fear consolidation and accelerates fear generalization. **A** Scheme of the experimental design. Vehicle or SCH58261 (0.1 mg/kg) were administered intraperitoneally (i.p.) immediately after contextual fear conditioning (CFC - 3 shocks of 0.7 mA). **B** Individual values and mean ± SEM (*n* = 9–11) of the percentage of time freezing in context A (paired with foot-shocks) or in the unpaired context B, at 1 and 2 days after conditioning, respectively. **C** Discrimination index of CFC animals at 1 and 2 days after conditioning; when probed for recent fear memory, SCH58261-injected rats had a lower discrimination index compared with the saline (control) group, showing a worst ability to distinguish between contexts A and B. **D** Scheme of the experimental design, where vehicle or SCH58261 (0.1 mg/kg) were administered i.p. immediately after a weak CFC protocol (1 shock of 0.5 mA). **E** Individual values and mean ± SEM (*n* = 10) of the percentage of time freezing in the paired context A or in the unpaired context B, at 1 and 2 days after CFC, respectively. **F** Discrimination index of rats subjected to a weak CFC, probed at 1 and 2 days after conditioning. Only the animals that were injected with SCH58261 after CFC discriminated between contexts A and B when probed for recent fear memory. **B**, **E** *p < 0.05 and ***p < 0.01 compared to the control group, treated with vehicle (two-way ANOVA followed by Fisher’s LSD multiple comparison test); **C**, **F** **p < 0.01, compared to the group treated with vehicle (Student’s *t* test) and ^#^*p* < 0.05 or ^##^*p* < 0.01, one sample *t* test comparing with the hypothetical value of 0.5 (i.e., no discrimination between contexts A and B).

**Fig. 2:**
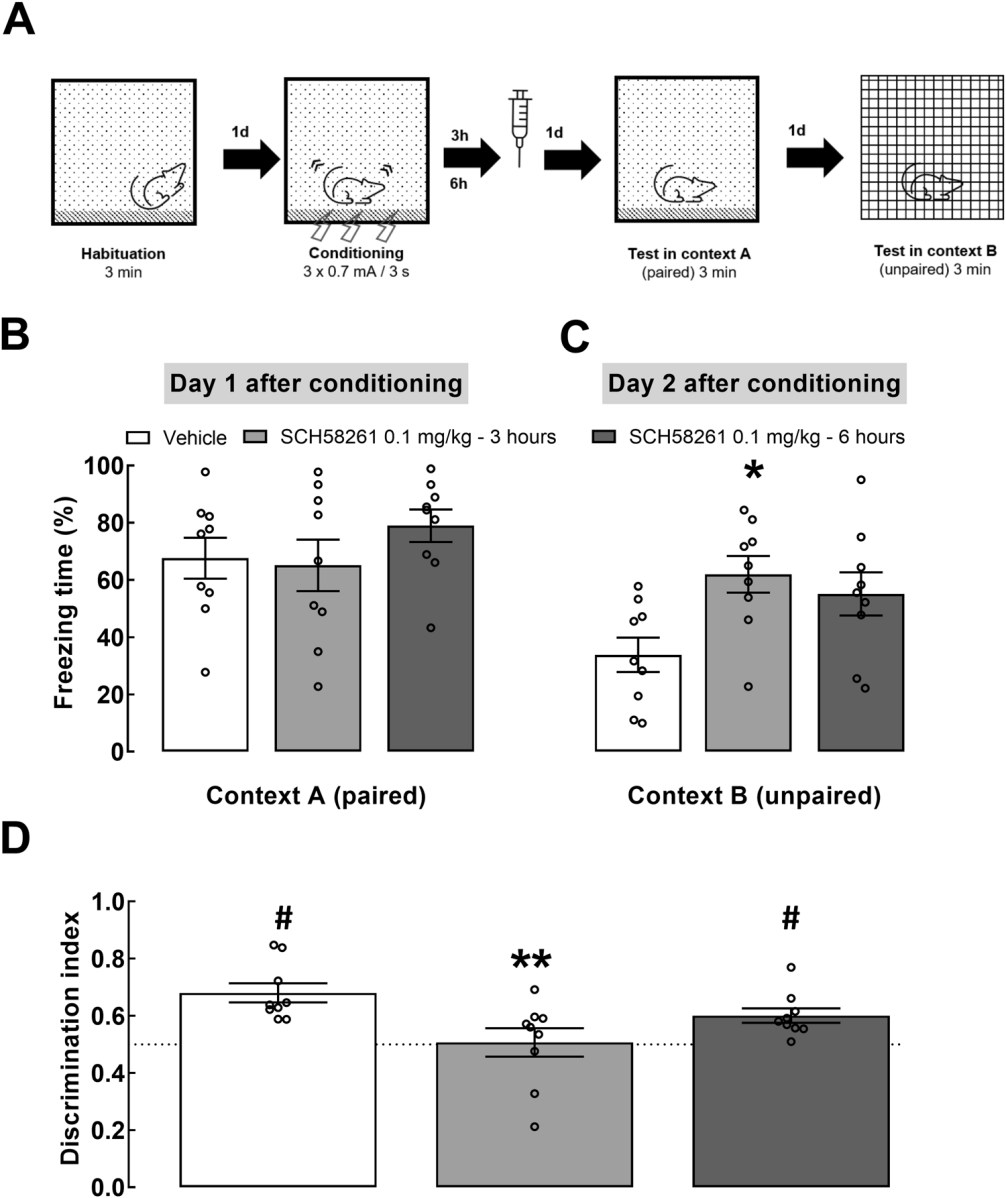
The blockade of A_2A_R accelerates fear generalization only when done during the early stages of memory consolidation. **A** Scheme of the experimental design. Vehicle or SCH58261 (0.1 mg/kg) were administered i.p. 3 or 6 h after contextual fear conditioning (CFC - 3 shocks of 0.7 mA). **B** Individual values and mean ± SEM (*n* = 9) of the percentage of time freezing in context A (paired with foot-shocks), 1 day after conditioning. **C** Individual values and mean ± SEM (*n* = 9) of the percentage of time freezing in the unpaired context B at 2 days after conditioning. **D** Discrimination index probed at 1 and 2 days after conditioning; animals that were injected with SCH58261 3 h after CFC did not discriminate between contexts when probing for recent fear memory, unlike saline-treated animals and the rats injected with SCH58621 6 h after CFC. **B**, **C** *p < 0.05 compared to the control group treated with vehicle (one-way ANOVA followed by a Dunnett’s post hoc test); **D** ** *p* < 0.01 in relation to the control group treated with vehicle (one-way ANOVA followed by a Dunnett’s post hoc test) and #*p* < 0.05, one sample *t* test comparing with the hypothe*t*ical value of 0.5 (no discrimination between contexts A and B).

**Fig. 3:**
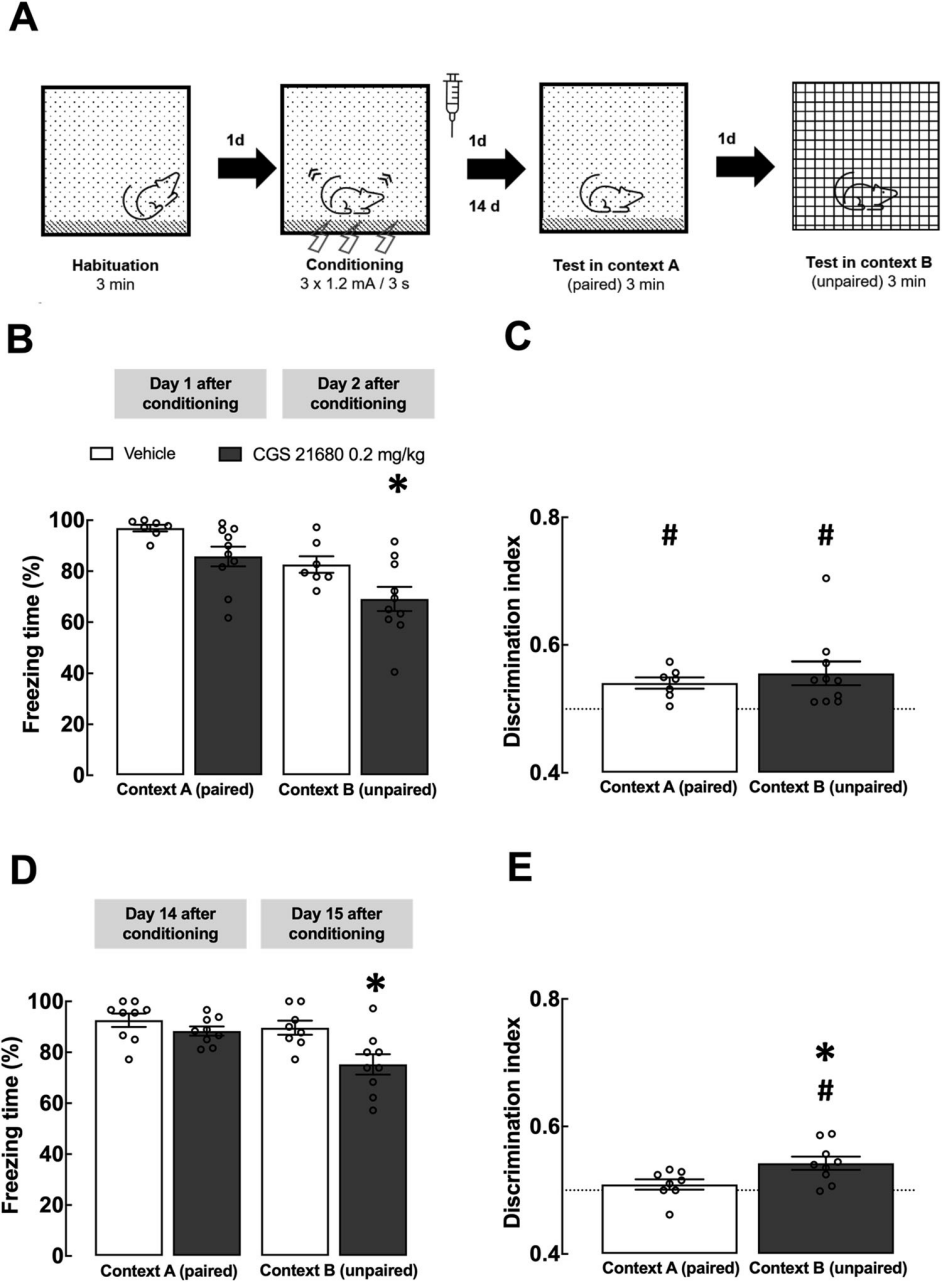
Activation of A_2A_R during fear consolidation delays fear generalization. **A** Scheme of the experimental design. Vehicle or CGS21680 (0.2 mg/kg) were administered i.p. immediately after a strong intensity fear conditioning session (3 shocks of 1.2 mA). **B** Individual values and mean ± SEM (*n* = 7–10) of the percentage of time freezing in the paired context A and in the unpaired context B at 1 and 2 days after conditioning, respectively. **C** Discrimination index at 1 and 2 days after fear conditioning; both groups of animals discriminate between contexts A and B, when probed for recent fear memory. **D** Percentage of time freezing in the paired context A and in the unpaired context B at 14 and 15 days after conditioning, respectively, in the same group of animals. **E** Discrimination index at 14 and 15 days after fear conditioning; animals that were injected with CGS21680 still discriminate between contexts, in contrast to the saline group, when probed for remote fear memory. **B**, **D** *p < 0.05 in relation to the group treated with vehicle (two-way ANOVA followed by Fisher’s LSD multiple comparison test); **C**, **E** *p < 0.05 compared to the control group treated with vehicle (Student’s *t* test) and ^#^*p* < 0.05, one sample *t* test when compared with the hypo*t*hetical value of 0.5 (no discrimination between contexts A and B).

**Fig. 4:**
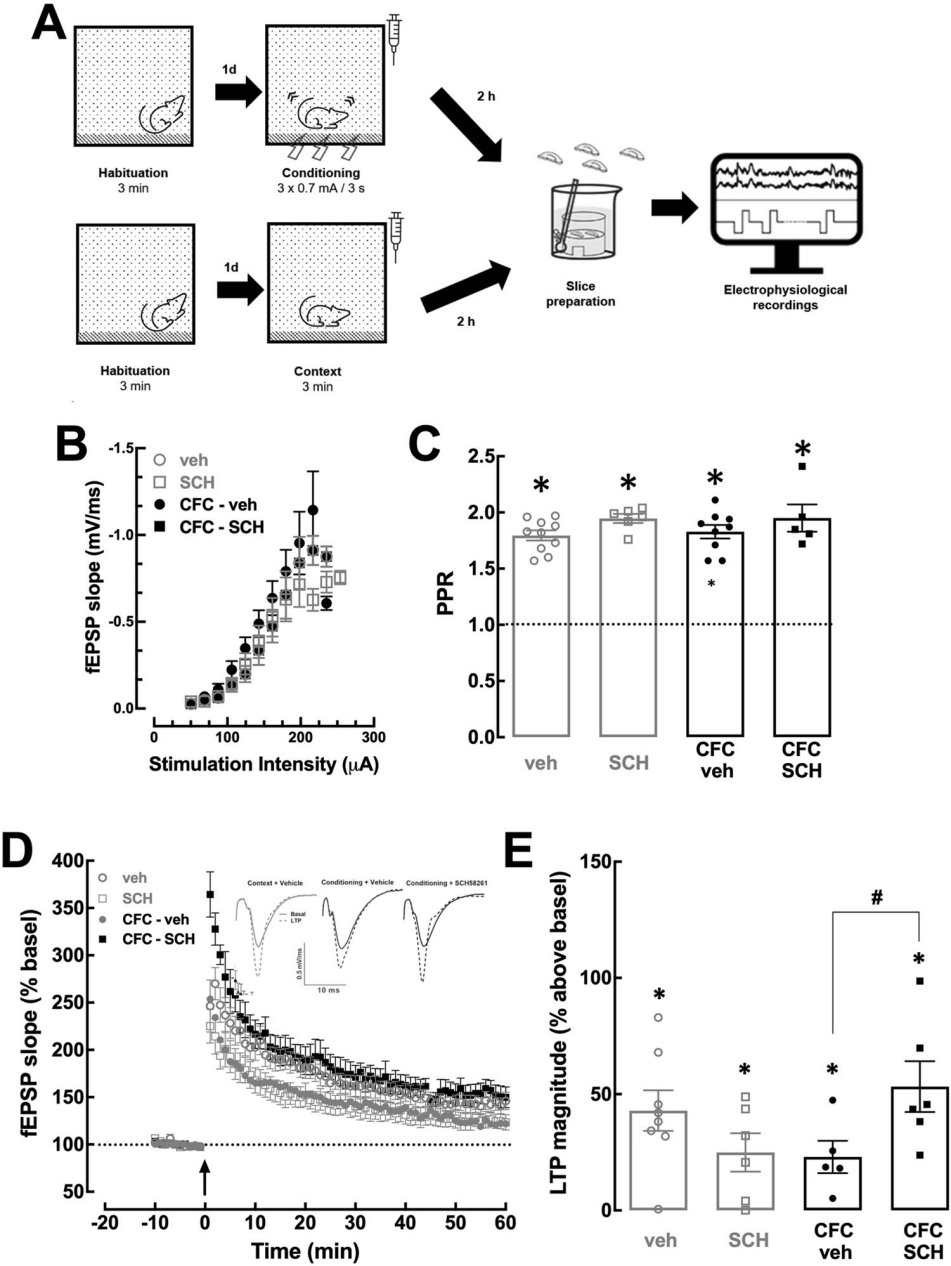
Blockade of A_2A_R immediately after contextual fear conditioning attenuates conditioning-induced decrease in long-term potentiation in the dorsal hippocampus. **A** Scheme of the experimental design. Animals were injected i.p. with vehicle or SCH58261 (0.1 mg/kg) immediately after CFC (3 shocks of 0.7 mA), corresponding to the groups labeled as CFC-veh (black circles) and CFC-SCH (black squares), or exposure to the conditioning chamber (context A) without application of electrical shocks, corresponding to the groups labeled as veh (gray circles) and SCH (gray squares). Electrophysiological recordings (field excitatory post-synaptic potential—fEPSP) were performed on slices of the dorsal hippocampus (DH) collected 2 h after the i.p. injections. **B** Input–output (I/O) curves at CA3-CA1 synapses of the DH showed no significant differences between the different experimental groups. **C** Paired pulse ratio (PPR) showed a paired-pulse facilitation with an interpulse interval of 50 ms with no differences between groups. **D** Time-course of the variation of the slope of fEPSPs, expressed as percentage of baseline values, in the CA1 *stratum radiatum* upon stimulation of afferent Schaffer collateral fibers, before and after induction of a long-term potentiation (LTP) with a train of high-frequency stimulation (HFS, 1 train of 100 Hz for 1 s, arrow). The inserts show representative recordings of the fEPSPs obtained for the indicated experimental groups, prior to LTP induction (filled traces) and 50–60 min after LTP induction (dashed lines). **E** Bar graph of LTP magnitude at 50–60 min after HFS. The blockade of A_2A_R after CFC prevented the decrease of LTP magnitude induced by CFC in the DH. Individual values and mean ± SEM (*n* = 5–10) are presented in the bar graphs. *p < 0.05 in relation to the value of 1 (**C**) or 0 (**E**) (one sample *t* test, when comparing with a hypothetical value); ^#^*p* < 0.05 between the indicated groups (two-way ANOVA and Fisher’s LSD multiple comparison test).

The total duration of the experimental protocol was variable, according to the experiment (Figs. 1A, D, 2A, 3A, 4A and Supplementary Fig. 1A, D, G), with a maximum duration of 15 days, testing only the generalization of contextual fear without any tones being applied: in context A (paired with electrical stimulation) and context B (where animals were never shocked, i.e., unpaired context). On day 1 after conditioning, the animals were re-exposed to the box paired with the electrical stimuli in the paws (paired context A) for 3 min (except in the experiment schematized in Supplementary Fig. 1D, in which animals were re-exposed to context A on day 2 after fear conditioning), aiming at evoking aversive memory (fear retrieval) and to evaluate the responses of conditioned fear (i.e., freezing). On day 2 after conditioning, the animals were exposed for 3 min to a box (30 × 30 × 30 cm) with glass walls and floor and gridded ceiling where the animals were never shocked (unpaired context B), except in the experiment schematized in Supplementary Fig. 1D, in which rats were exposed to context B on day 1 after fear conditioning. The same animals were re-exposed 14 and 15 days after contextual fear conditioning (CFC) to contexts A and B, respectively, in experiments summarized in Fig. 3 and Supplementary Fig. 1A. The time spent freezing, defined as the absence of movements except those necessary for breathing and vocalization, was measured as an expression of fear and as a memory retention index. During the exposure(s) to the paired context (A) or to the unpaired context (B), the freezing time was quantified (in seconds) every minute and was expressed as a percentage of the total time of the experimental session. To evaluate contextual fear generalization, a discrimination index was calculated as the relative freezing behavior of rats in both contexts, according to the following formula: discrimination index= (training context)/(training context + novel environment). A ratio of 1 indicates that rats were able to discriminate the contexts perfectly, and a ratio of 0.5 or less means that the animals were unable to discriminate between contexts [28]. The experiments were video recorded, allowing the experimenter to remain in another room, and monitor the animal’s behavior throughout, as well as blindly ranking behavior. The experimental sessions were performed under 20 lux luminosity. The cleaning of the contexts was done using a 10% ethanol solution between the exposure of each animal.

### Electrophysiological recordings

Two hours after CFC using the intermediate intensity protocol (0.7 mA foot-shocks), rats were anesthetized under a halothane atmosphere and sacrificed by decapitation. The brain was quickly removed and placed in ice-cold artificial cerebrospinal fluid (aCSF) containing (in mM); 124 NaCl, 4.5 KCl, 2 CaCl_2_, 1 MgCl_2_, 26 NaHCO_3_, 1.2 NaH_2_PO_4_ and 10 D-glucose, bubbled with a gas mixture of 95% O_2_ and 5% CO_2_. The brains were sectioned into 400 µm thick horizontal slices (cut from the ventral towards the dorsal part of the brain) with a vibratome (Vibratome 1500, Leica, Wetzlar, Germany) for the preparation of amygdala slices. Then, to prepare hippocampal slices, the hippocampi were isolated and transverse slices, 400 μm thick, were prepared using a McIlwain tissue chopper (Campden Instruments, UK). Slices were then transferred to an incubation chamber filled with gassed aCSF and allowed to recover for 1 h at 32.0 °C before being transferred to a recording chamber (1 mL capacity) and continuously superfused with gassed aCSF, kept at 30.5 °C, at a constant rate of 3 mL/min. The stimulation of the slices was delivered every 20 s with 0.1 millisecond rectangular pulses at 0.05 Hz under basal conditions through a concentric bipolar stainless steel electrode connected to a S44 electrical stimulator (Grass Instruments, West Warwick, RI, USA) and the recording electrode consisted in a micropipette filled with 4 M NaCl (2–4 MΩ resistance).

In slices from the dorsal hippocampus (DH), the stimulation electrode was placed over the Schaffer collateral-commissural pathway and the recording electrode was placed in the *stratum radiatum* of the CA1 area. The orthodromically evoked field excitatory postsynaptic potentials (fEPSPs) were recorded, amplified using an ISO-80 amplifier (World Precision Instruments, Hertfordshire, UK), and digitized using an analog-digital converter ADC-42 board (Pico Technologies, Pelham, NY, USA). The postsynaptic response was quantified as the maximum slope of the rising phase of the fEPSPs and three consecutive responses were continuously averaged and monitored on a PC-type computer using the WinLTP 1.01 software (WinLTP, RRID:SCR_008590) [29]. To evaluate basal neurotransmission, input/output curves were first acquired by continuously increasing the current of the stimulus and measuring the slope of the evoked response, starting with a current that elicited no response and terminating when the response stabilized or when the fEPSP was contaminated by a population spike. Based on the input-output curves, a stimulus that evoked a signal of circa 40% of the maximal slope was chosen. The paired-pulse ratio (PPR) was investigated by applying two pulses with an interpulse interval of 50 milliseconds. LTP was induced by applying a high frequency stimulation (HFS) consisting of a single train of pulse at 100 Hz for 1 s [24, 25]. The magnitude of LTP was evaluated by comparing the average of the fEPSP slopes from 50 to 60 min after HFS with the average of the fEPSP slopes 10 min before the HFS (baseline) and is represented as percentage of change from baseline.

**Fig. 5:**
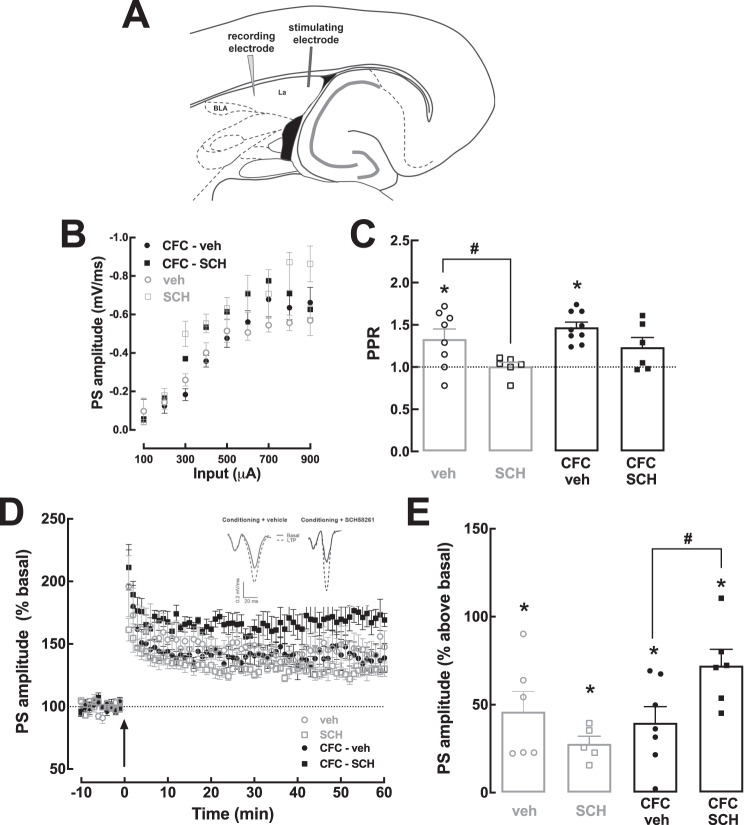
The blockade of A_2A_R immediately after contextual fear conditioning increases long-term potentiation in the lateral amygdala. **A** Scheme of slices containing the amygdala showing the position of the electrodes for extracellular electrophysiological recordings of population spikes (PS) at the lateral amygdala (LA). The animals received vehicle or SCH58261 (0.1 mg/kg), i.p., immediately after CFC (3 shocks of 0.7 mA), corresponding to the groups labeled as CFC-veh (black circles) and CFC-SCH (black squares), or exposure to the conditioning chamber (context A) without application of electrical shocks, corresponding to the groups labeled as veh (gray circles) and SCH (gray squares). Electrophysiological recordings were performed on horizontal slices containing the lateral nuclei of the amygdala (LA), collected 2 h after the i.p. injections. **B** Input-output (I/O) curves at LA excitatory synapses showed no significant differences between the different experimental groups. **C** Paired pulse ratio (PPR) showed that a paired-pulse facilitation (with an interpulse interval of 30 ms) in vehicle-injected groups, which was abrogated after exposure to SCH58261, regardless of CFC. **D** Time-course of the variation of the amplitude of PS (expressed as percentage of baseline values) in the LA before and after induction of LTP with high-frequency stimulation (HFS, 3 trains of 100 Hz for 1 s, with 5 s inter-train interval). The inserts show representative recordings of the PS obtained for the indicated experimental groups, prior to LTP induction (filled traces) and 50–60 min after LTP induction (dashed lines). **E** Bar graph of LTP magnitude at 50–60 min after HFS. The blockade of A_2A_R immediately after CFC increased LTP magnitude when compared with animals subjected to CFC and treated with vehicle. Values are expressed as mean ± SEM of *n* = 5–9 rats; * indicates a significant difference (*p* < 0.05) in relation to the value of 1 for **C** or 0 for **E** (one sample *t* test, comparing with a hypothetical value). # indicates a significant difference (*p* < 0.05) between the indicated groups, observed with two-way ANOVA followed by Fisher’s LSD multiple comparison test.

In amygdala slices, both the stimulus and the recording electrodes were placed in the lateral nuclei of the amygdala (LA), as represented in Fig. 5A. The post-synaptic response was measured as the amplitude of population spikes (PS), quantified as the distance from the maximal negative peak of the PS to a line tangent to the lower and upper positive shoulders of the PS. Input-output curves were acquired as described above by continuously increasing the current applied by the stimulus electrode, starting with a current that elicited no response and terminating when the evoked PS amplitude stabilized. Again, the input-output curve directed the choice of a stimulus intensity that evoked a signal of circa 40% of the maximal PS amplitude. PPR was investigated by applying two pulses with an interpulse interval of 30 milliseconds. LTP was induced by HFS consisting of three trains of pulses at 100 Hz delivered with a 5 s interval [19]. The magnitude of LTP was calculated by comparing the average of PS amplitudes 50–60 min after HFS with the average of the PS amplitude 10 min before the HFS (baseline). LTP values were represented as the percentage of change from the baseline.

### Data analysis and statistics

In all experimental procedures, results are presented as mean ± SEM from n samples (*n* = number of rats) together with the individual data for each animal. Animals were randomly assigned to the different groups and the estimate of the number of animals in each group was based on our previous experience on the variability of animal behavior and of electrophysiological responses linked to an expect size effect of drugs and treatments larger than 10% of control values. The experimenters were unaware to which group each rat belonged. Analyses were performed using Statistica 11® or GraphPad Prism 8.1.1. and the significance level was set at *p* values < 0.05. Normality was assessed using Shapiro–Wilk test. One sample Student’s *t* test (two-tailed) was used when comparing the mean of a sample from an experimental group with a pre-defined hypothetical value. Single statistical comparisons between two independent experimental groups following a normal distribution were analyzed using an unpaired Student’s *t* test, whereas one or a two-way analysis of variance (ANOVA) for independent means was used for comparisons between more than two groups, followed by Dunnett’s post hoc test, when comparing with the vehicle-treated group, or Fisher’s LSD multiple comparison test. The identification of outliers was carried out using a Grubbs’ test.

## Results

### Blockade of A_2A_R increases fear consolidation and accelerates fear generalization

Since fear consolidation influences fear generalization [9, 30], we first investigated the impact of blocking A_2A_R during fear consolidation on fear generalization. Rats were injected intraperitoneally (i.p.) with the A_2A_R selective antagonist SCH58261 (0.1 mg/kg) or vehicle, immediately after contextual fear conditioning (CFC, using an aversive stimulus of intermediate intensity: 3 × 0.7 mA foot-shocks/3 s). Animals were then re-exposed to the context paired with foot-shocks (context A), 1 and 14 days after CFC and to a novel/safe (unpaired) context (context B), 2 and 15 days after CFC (Fig. 1A; Supplementary Fig. 1A). Importantly, in this experiment as well as in all following CFC experiments, we always confirmed that the average freezing after fear conditioning acquisition and before the addition of any drug was never statistically different between the different groups in each experiment, thus ensuring that any modification caused by the administration of a drug corresponds to an effect of the tested drug in the processes of consolidation and generalization (see Supplementary Fig. 2). As expected, vehicle-treated animals froze more in context A than in context B, 1 and 2 days after CFC, respectively (context A: 62.9 ± 7.0%; context B: 32.4 ± 56.0% freezing, *p* = 0.0039, t test; Fig. 1B). This no longer occurred 14 and 15 days after CFC (context A: 63.6 ± 7.6%; context B: 51.5 ± 8.6%, *p* = 0.31, t test; Supplementary Fig. 1B), as observed in a different group of rats.

A discrimination index (DI) was used (see Methods) to quantify fear generalization, where DI > 0.5 indicates discrimination between contexts A and B [28]. Vehicle-treated rats displayed a DI = 0.71 ± 0.04 at 1–2 days post-CFC (*p* < 0.001, one sample *t* test; Fig. 1C), as expected for recent fear memory [28]. At 14–15 days post-CFC, animals did not discriminate between contexts, showing fear generalization (DI = 0.57 ± 0.04, *p* = 0.166, one sample *t* test; Supplementary Fig. 1C), as described for labile remote fear memory [9, 28]. However, SCH58261-treated rats increased freezing in both contexts at 1–2 days post-CFC: thus, SCH58261 significantly modified the freezing behavior in both contexts A and B (context A, 1 day post-CFC: SCH58261 84.0 ± 4.2% and vehicle 62.9 ± 7.0%, *p* = 0.012; context B, 2 days post-CFC: SCH58261 62.9 ± 5.3% and vehicle 32.4 ± 5.9%, *p* < 0.001; Fig. 1B), as assessed with a two-way ANOVA (effect of context F_1,19_ = 46.39, *p* < 0.001; effect of SCH58261 F_1,19_ = 13.54, *p* = 0.002; interaction F_1,19_ = 0.854, *p* = 0.233); moreover, both groups discriminated between contexts (SCH58261: DI = 0.57 ± 0.015, *p* = 0.002; vehicle: DI = 0.72 ± 0.04, *p* = 0.002; Fig. 1C). Interestingly, SCH58261-treated rats displayed decreased DI compared to controls (*p* = 0.004; Fig. 1C).

When a different group of animals (which underwent a similar CFC manipulation and drug treatment as these described above), were re-exposed to contexts A and B 14 and 15 days later, respectively, SCH58261-treated animals maintained higher fear responses than controls: thus, SCH58261 significantly modified the freezing behavior in both contexts A and B (context A, 14 day post-CFC: vehicle 63.6 ± 7.6% and SCH58261 88.7 ± 2.7%, *p* = 0.005) and in context B, 15 days post-CFC: vehicle 51.5 ± 8.6% and SCH58261: 79.1 ± 3.7%, *p* = 0.003; Supplementary Fig. 1B), as assessed with a two-way ANOVA (effect of context F_1,15_ = 5.084, *p* = 0.040; effect of SCH58261 F_1,15_ = 14.84, *p* = 0.002; interaction F_1,15_ = 0.069, *p* = 0.797); however, both experimental groups did not discriminate between contexts (SCH58261: DI = 0.53 ± 0.01, *p* = 0.083; vehicle: DI: 0.57 ± 0.04, *p* = 0.166; Supplementary Fig. 1C). Thus, A_2A_R blockade during fear consolidation increased both recent and remote fear memory but decreased memory accuracy/specificity, thereby promoting fear generalization.

To further test if A_2A_R blockade after CFC increased fear consolidation affecting the accuracy of fear memory, a different group of rats were CFC using a weak unconditioned stimulus (1 × 0.5 mA foot-shock, 3 s; Fig. 1D), which yields poorer fear acquisition and memory and consequent lack of discrimination between paired and unpaired contexts [31]. Here, SCH58261-treated animals showed improved fear memory in context A (SCH58261: 57.2 ± 6.9%; vehicle: 35.95 ± 4.6%, *p* = 0.007; Fig. 1E) and discriminated between contexts (SCH58261: DI = 0.60 ± 0.04, *p* = 0.038), unlike vehicle-treated rats (DI = 0.53 ± 0.03, *p* = 0.327; Fig. 1F). No significant differences were observed between groups in context B, 2 days after CFC (vehicle: 30.6 ± 3.0%; SCH58261: 38.1 ± 5.5%; *p* = 0.312; Fig. 1E). Thus, A_2A_R blockade after CFC increases memory specificity after weak fear learning but decreasing memory specificity after stronger fear learning.

To determine if the order of exposure to contexts A and B influences fear memory and SCH58261 effects, we repeated CFC using foot-shocks of intermediate intensity but now animals were exposed to context B on day 1 post-CFC and to context A on day 2 post-CFC (Supplementary Fig. 1D). Two-way ANOVA identified an interaction between context and SCH58261 (F_1,16_ = 5.02, *p* = 0.039) and Fisher’s LSD test showed larger freezing in context B of SCH58261- *versus* vehicle-treated animals (SCH58261: 69.4 ± 5.1%; vehicle: 45.1 ± 7.5%; *p* = 0.025) without significant differences between groups in context A (SCH58261: 62.8 ± 8.3%; vehicle: 64.0 ± 8.0%, *p* = 0.911) (Supplementary Fig. 1E). Furthermore, vehicle-treated animals discriminated between contexts (DI = 0.59 ± 0.03, *p* = 0.016) but SCH58261-treated animals did not (DI = 0.46 ± 0.04, *p* = 0.336) (Supplementary Fig. 1F) (t_16_ = 2.73, *p* = 0.015 between groups). Thus, the order of context presentation after CFC does not influence fear generalization or the effect of A_2A_R blockade thereupon, although the potentiating effect of SCH58261 on fear memory strength (in the paired context) seems absent when the paired context is tested second (confront data in Supplementary Fig. 1F with data presented in Fig. 1B).

Finally, to investigate if A_2A_R blockade after CFC specifically affected associative fear memory rather than affecting the sensitization to defensive responses, another group of animals were treated with either vehicle or SCH58261 after receiving immediate foot-shocks in context A, to avoid the association between context and aversive stimulus (Supplementary Fig. 1G). A two-way ANOVA did not reveal statistical differences in both contexts (F_1,16_ = 0.073, *p* = 0.790) nor an effect of SCH58261 (F_1,16_ = 0.072, *p* = 0.792). Moreover, neither groups discriminated between contexts (vehicle: DI = 0.45 ± 0.06, *p* = 0.423; SCH58261: DI = 0.46 ± 0.07, *p* = 0.640) with no differences between groups (*t*_16_ = 0.148, *p* = 0.884). Thus, the association between the context-foot-shocks only happens when animals first learn about contextual cues before receiving the foot-shocks and show that SCH58261 specifically affects associative fear memory.

### The blockade of A_2A_R accelerates fear generalization only when it happens early on in the consolidation time-window

To assess if A_2A_R blockade during later stages of CFC also affects fear retrieval and generalization, rats were injected with either vehicle or SCH58261 (0.1 mg/kg), 3 or 6 h after CFC (Fig. 2A). No differences were observed in fear response to context A, 1 day post-CFC (vehicle: 67.6 ± 7.2%; SCH58261 at 3 h: 65.1 ± 9.0%; SCH58261 at 6 h: 79.0 ± 5.7%, F_2,24_ = 0.99, *p* = 0.38; Fig. 2B). However, SCH58261-treated animals displayed increased freezing in context B, 2 days post-CFC (vehicle: 33.8 ± 6.0%; SCH58261 at 3 h: 62.0 ± 6.4%; SCH58261 at 6 h: 55.1 ± 7.5%, F_2,24_ = 4.80, *p* = 0.020, one-way ANOVA). Interestingly, compared to vehicle-treated, rats treated with SCH58261 3 h post-CFC froze significantly more in context B (*p* = 0.011) but there was only a tendency to increased freezing when animals were treated with SCH58261 6 h post-CFC (*p* = 0.062, Dunnett´s post hoc test; Fig. 2C). Furthermore, both vehicle-treated and rats treated with SCH56281 6 h post-CFC discriminated between contexts (vehicle: DI = 0.68 ± 0.03, *p* < 0.001; SCH58261 6 h post-CFC: DI = 0.60 ± 0.03, *p* = 0.004) whereas animals treated with SCH58261 3 h post-CFC did not (DI: 0.51 ± 0.05, *p* = 0.894). One-way ANOVA with Dunnett’s post hoc test showed a significant effect of the timing of SCH58261 treatment (F_2,24_ = 5.342, *p* = 0.012) and a difference in DI between vehicle- and SCH58261-treated rats 3 h post-CFC (*p* = 0.006) but not between control and SCH58261-treated animals 6 h post-CFC (*p* = 0.253). Thus, A_2A_R blockade only decreases the accuracy of contextual fear memory when it occurs early on during fear consolidation, indicating that A_2A_R have a prominent role in early mechanisms of memory consolidation.

### Activation of A_2A_R during fear consolidation decreases fear generalization

To investigate if A_2A_R activation during fear memory consolidation could decrease fear generalization, in contrast to the effect of the A_2A_R antagonist, rats were i.p.-treated either with vehicle or with the selective A_2A_R agonist CGS21680 (0.2 mg/kg), immediately after CFC, using a strong CFC protocol (3 × 1.2 mA foot-shocks, 3 s; Fig. 3A). This strong CFC protocol was selected based on the rationale that pathological conditions of fear consolidation and generalization that need to be therapeutically controlled, are more frequent following intense emotional challenges; although such a choice avoids a possible floor effect with CGS21680, it simultaneously increases the likeliness of a possible ceiling effect in the vehicle-treated group, making it potentially more difficult to assess the magnitude of generalization in the control group. Two-way ANOVA revealed a significant effect of both context (F_1,15_ = 21.77, *p* < 0.001) and CGS21680 (F_1,15_ = 7.345, *p* = 0.016): CGS21680-treated animals had a tendency to freeze less in context A, 1 day post-CFC (vehicle: 96.9 ± 1.3%; CGS21680: 85.8 ± 3.9%; *p* = 0.057, Fisher’s LSD test) and froze less in context B, 2 days post-CFC (vehicle: 82.6 ± 3.3%; CGS21680: 69.1 ± 4.7%; *p* = 0.023; Fig. 3B). However, both groups discriminated between contexts (vehicle: DI = 0.54 ± 0.01, *p* = 0.004; CGS21680: DI = 0.55 ± 0.02, *p* = 0.015) with no differences between groups (*t*_15_ = 0.643, *p* = 0.530; Fig. 3C). When probing in the same group of animals for remote fear memory at 14–15 days post-CFC (the freezing behavior of one rat treated with CGS21680 forced its exclusion as concluded using the Grubbs’ test), there was a significant interaction between context and CGS21680 treatment (F_1,15_ = 5.366, *p* = 0.035): CGS21680-treated animals froze less in context B (vehicle: 89.6 ± 2.8%; CGS21680: 75.3 ± 4.0%; *p* = 0.012) but there were no differences between groups in context A (vehicle: 92.6 ± 2.6%; CGS21680: 88.3 ± 1.8%; *p* = 0.197; Fig. 3D). Interestingly, CGS21680-treated animals still discriminated between contexts at this time point (DI = 0.54 ± 0.01, *p* = 0.004) unlike vehicle-treated rats (DI = 0.51 ± 0.01, *p* = 0.293) (t_15_ = 2.47, *p* = 0.026 between groups). Thus, A_2A_R activation during fear consolidation decreases fear generalization.

### A_2A_R blockade immediately after CFC reverts conditioning-induced decrease in hippocampal long-term potentiation (LTP) and increases LTP in the lateral amygdala of conditioned rats

Since consolidation of contextual fear memory is considered to involve synaptic plasticity mechanisms in both lateral amygdala (LA) and dorsal hippocampus (DH) [4, 8] and A_2A_R selectively control synaptic plasticity in these brain regions [19, 25], we tested if the effects of SCH58261 on fear memory consolidation were associated with altered synaptic plasticity in CA3-CA1 synapses of DH and/or excitatory synapses of LA in rats sacrificed 2 h after i.p. injections, which were performed immediately after context exposure or CFC (Fig. 4A).

Input/output curves did not reveal alterations of basal synaptic transmission in the different groups, in both DH (Fig. 4B) and LA (Fig. 5B). In DH, all groups displayed a similar paired-pulse ratio (PPR): a two-way ANOVA indicated no effect of CFC (F_1,26_ = 0.08; *p* = 0.78) or of SCH58261 (F_1,26_ = 4.22; *p* = 0.05), nor an interaction between SCH58261 and CFC (F_1,26_ = 0.05; *p* = 0.82) (Fig. 4C). This confirms our previous results [25] that SCH58261 does not influence the probability of neurotransmitter release and short-term plasticity in DH, even after CFC.

In LA, there was a paired pulse facilitation (i.e., PPR > 1), in control (i.e. non-CFC)+vehicle (PPR = 1.33 ± 0.12, *t*_7_ = 2.81; *p* = 0.03, one sample t test) and in CFC+vehicle (PPR = 1.47 ± 0.06, *t*_8_ = 7.82, *p* < 0.0001) rats, but not in control+SCH58261 (PPR = 1.01 ± 0.05, *t*_5_ = 0.17; *p* = 0.87) nor in CFC + SCH58261 (PPR = 1.23 ± 0.11, *t*_5_ = 2.06, *p* = 0.09) rats (Fig. 5C). A two-way ANOVA confirmed an effect of SCH58261 (F_1,25_ = 9.2; *p* = 0.006) and Fisher’s LSD test confirmed a lower PPR in SCH58261-treated rats (*p* = 0.02). Thus, SCH58261 increases the probability of neurotransmitter release and abolishes paired pulse facilitation in LA excitatory synapses, whereas CFC does not alter PPR in LA (Fig. 5C).

HFS consistently induced LTP in DH in all groups (Figs. 4D, E). A two-way ANOVA showed an interaction between CFC and SCH58261 (F_1,20_ = 9.92; *p* = 0.005) and a Fisher’s LSD test indicated a decreased LTP amplitude in CFC+vehicle (LTP magnitude 23.0 ± 6.9% over baseline) compared to control+vehicle rats (49.0 ± 7.2%, *p* = 0.047) or when compared to CFC + SCH58261 (53.3 ± 10.9%, *p* = 0.03). Thus, A_2A_R blockade immediately after CFC restored LTP amplitude in DH to values similar to control animals.

HFS consistently induced LTP in LA in all groups (Figs. 5D, E). A two-way ANOVA showed an interaction between CFC and SCH58261 (F_1,20_ = 7.2; *p* = 0.01) and a Fisher’s LSD test showed an increased LTP magnitude in CFC + SCH58261 (72.1 ± 9.3% above baseline) compared to CFC+vehicle rats (39.7 ± 9.2%, *p* = 0.02). Thus, A_2A_R blockade immediately after CFC increased LTP in LA.

### The altered A_2A_R-mediated control of CFC-induced abnormal hippocampal LTP is mostly independent of adenosine A_1_ receptors

Adenosine modulation involves a coordinated action of A_2A_R and adenosine A_1_ receptors (A_1_R) [10, 32]. Accordingly, there is a tight A_1_R/A_2A_R interplay in different brain areas, involving a combination of direct A_1_R-A_2A_R interaction/heteromerization [33–36] and circuit-mediated effects [37–39]. This prompts testing if the altered A_2A_R effects after CFC are secondary to putative alterations of A_1_R function, which are associated with modifications of mood and memory [40]. Thus, we investigated if CFC alters A_1_R-mediated modulation of synaptic transmission in DH and LA, two areas proposed to be associated with fear generalization after CFC.

**Fig. 6:**
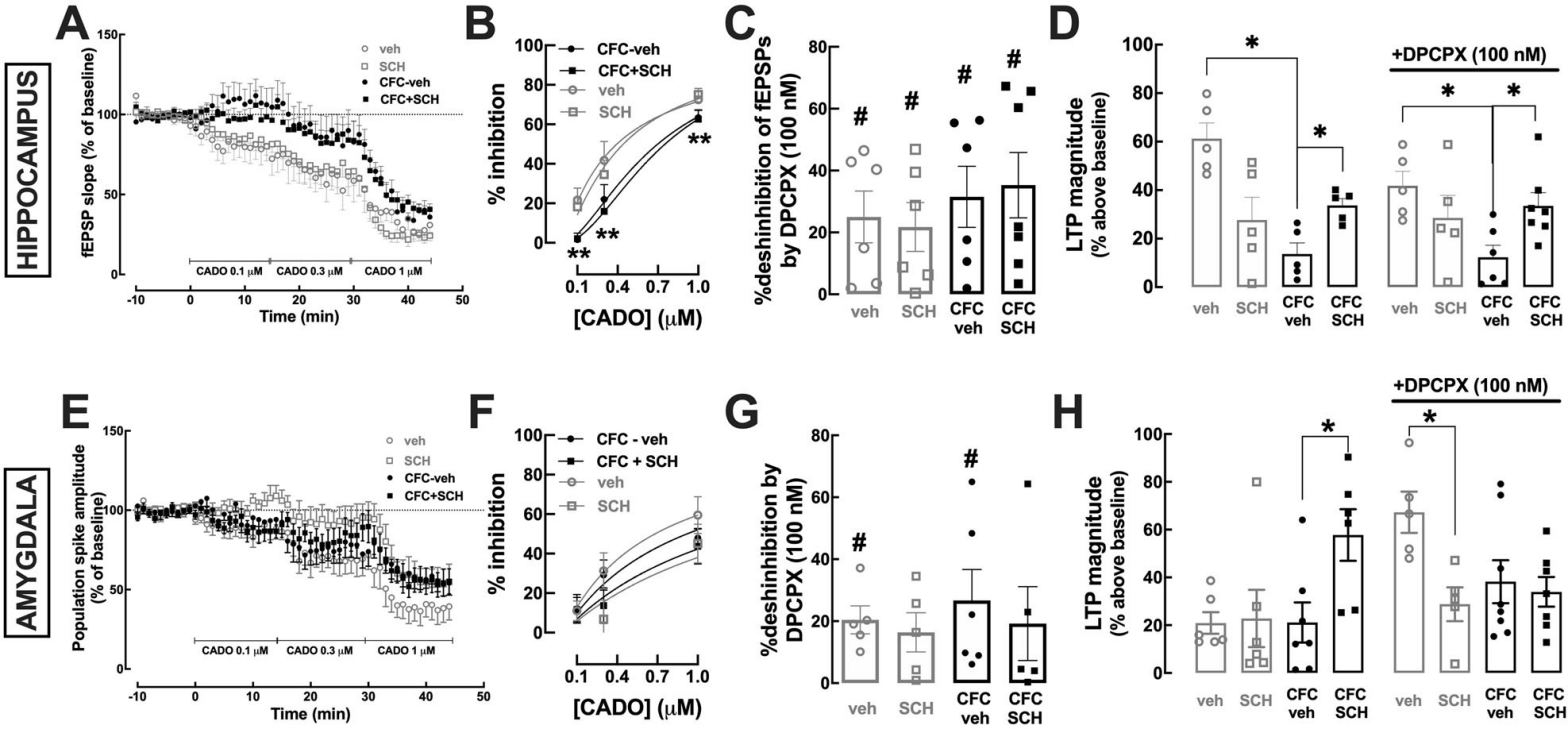
The ability of blocking A_2A_R immediately after contextual fear conditioning to normalize the increased long-term potentiation are independent of A_1_R in the dorsal hippocampus but not in the lateral amygdala. **A**, **E** Time-course of the variation, expressed as percentage of baseline values, of the slope of field excitatory post-synaptic potentials (fEPSPs) in the CA1 *stratum radiatum* upon stimulation of the afferent Schaffer collateral fibers (**A**) or of the amplitude of population spikes in synapses of the lateral amygdala (**E**), upon exposure to increasing concentrations of 2-chloroadenosine (CADO) in slices of rats receiving vehicle or SCH58261 (0.1 mg/kg), i.p., immediately after contextual fear conditioning (CFC - 3 shocks of 0.7 mA), corresponding to the groups labeled as CFC-veh (black circles) and CFC-SCH (black squares), or exposure to the conditioning chamber (context A) without application of electrical shocks, corresponding to the groups labeled as veh (gray circles) and SCH (gray squares). **B**, **F** The CADO concentration-dependent inhibition of fEPSPs or population spikes (and the fitted sigmoids constrained at 0 and 100%) indicate a lower efficiency of A_1_R after CFC, irrespective of the exposure to SCH58261 in the DH (**B**), but no modification of A_1_R efficiency in the LA (**E**). **C**, **G** The disinhibition by the A_1_R antagonist, DPCPX (100 nM), of synaptic transmission is not modified by CFC or SCH58261 in the DH (**C**), whereas it is abolished by SCH58261 exposure in the LA, irrespective of CFC (**G**). **D**, **H** LTP magnitude in the DH was reduced by CFC and restored by exposure to SCH58261, irrespective of the absence of presence of DPCPX (**D**), whereas in the LA the magnitude of LTP was increased by exposure to SCH58261 in the absence, but not in the presence of DPCPX (**H**). Values are expressed as mean ± SEM of *n* = 5–7 rats; * indicates a significant difference (*p* < 0.05) in relation to the value of 1 for **C** (one sample *t* test, comparing with a hypothetical value). # indicates a significant difference (*p* < 0.05) between the indicated groups, observed with two-way ANOVA followed by Fisher’s LSD multiple comparison test.

As shown in Fig. 6A, B, in DH, CFC decreased A_1_R-mediated inhibition of synaptic transmission triggered by 2-chloroadenosine (CADO; see [22]) compared to control rats (F_1,22_ = 6.04, *p* = 0.022), irrespective of SCH58261 (0.1 mg/kg) treatment (F_1,22_ = 21.02, *p* = 0.0001). However, there were no modifications of tonic A_1_R activation controlling synaptic transmission between groups, as concluded by similar effects of the A_1_R antagonist DPCPX (100 nM) (F_3,21_ = 0.44, *p* = 0.73) (Fig. 1C). Moreover, the impact of SCH58261 treatment on DH-LTP was not altered by DPCPX (Fig. 1D), with a similar pattern of decreased CFC-induced LTP deficits and recovery by SCH58261 both in the absence (F_1,16_ = 10.84, *p* = 0.0046) and presence of DPCPX (F_1,19_ = 7.1, *p* = 0.015). Thus, alterations of A_1_R-mediated function are not responsible for the ability of A_2A_R to correct CFC-associated abnormal LTP in DH.

In LA, there were no modifications of CADO-induced A_1_R-mediated inhibition of synaptic transmission between control and CFC animals (F_6,40_ = 1.7, *p* = 0.15) (Fig. 1E, F), nor of tonic A_1_R activation controlling synaptic transmission (F_3,32_ = 0.42, *p* = 0.53) (Fig. 1G). However, DPCPX increased LA-LTP magnitude only in control rats (*p* = 0.0007), an effect abrogated in SCH58261-treated rats (*p* = 0.68) (Fig. 1H), whereas the ability of SCH58261 treatment to increase LTP magnitude after CFC observed in the absence of DPCPX (*p* = 0.02) was abrogated by DPCPX (*p* = 0.70) (Fig. 1H). Thus, A_2A_R-mediated effects in LA depend on A_1_R function irrespective of CFC, probably due to the peculiar pharmacology of adenosine receptors [41] and/or different circuit-mediated A_1_R/A_2A_R interactions in this brain region [38] compared to other brain regions, namely to DH.

## Discussion

The present work shows that A_2A_R control the consolidation of context fear memory, impacting fear generalization. More specifically, it was shown that A_2A_R blockade immediately after contextual fear conditioning (CFC) bolsters and, conversely, A_2A_R activation limits, fear generalization. This effect of A_2A_R blockade is associated with a reversion of CFC-induced decrease of long-term potentiation (LTP) in dorsal hippocampus (DH) and with an increase of LTP in lateral amygdala (LA) after CFC.

Fear generalization is an adaptive process and refers to the emergence of fear responses in contexts not associated with previous negative experiences. Fear overgeneralization, however, is a maladaptive process characteristic of fear-related disorders such as PTSD, hampering fear extinction and the clinical management of these disorders [42]. Previous works showed that fear overgeneralization can result from abnormal consolidation of fear memory [9, 30]. Thus, unveiling mechanisms interfering with consolidation and subsequent generalization of fear is paramount to develop therapeutic strategies to control these maladaptive processes. We now show that an A_2A_R antagonist and agonist bidirectionally modulate fear memory consolidation and generalization. Thus, the selective A_2A_R antagonist SCH58261 increased retrieval of contextual fear memory in both the context paired with foot-shocks (context A) but also in an unpaired/safe context (context B), at both 1–2 days after CFC (i.e., recent memory) and 14–15 days post-CFC (i.e., remote fear memory), when using a mild intensity (3 × 0.7 mA) foot-shock protocol. This was accompanied with a decrease discrimination index (DI) when probing for recent memory. This suggests that SCH58261 accelerated fear generalization since this phenomenon only occurs at later time points, at remote memory retrieval [9]. In fact, when animals were CFC using a weak protocol (1 × 0.5 mA foot-shock), which leads to poor fear acquisition and memory [31], SCH58261 improved fear learning and DI of recent memory. These results also confirm that fear consolidation modulates the accuracy of fear memory therefore impacting on fear generalization, as previously shown [9, 30]. Moreover, our results show that the effects of the A_2A_R antagonist on fear memory and generalization specifically depend on mechanisms occurring during memory consolidation, since SCH58261 impaired contextual discrimination only when administered immediately after CFC or until 3 h later, having no effect on DI when injected 6 h after CFC, i.e., outside the consolidation time-window [43]. Accordingly, previous studies on the time-window of memory consolidation, showed that interfering with consolidation was less effective if done 1 h or more after memory acquisition [44].

Conversely, the selective A_2A_R agonist, CGS21680 decreased freezing in an unpaired/safe context and improved DI at remote memory retrieval: rats injected with CGS21680 after CFC, discriminated between the two contexts 14–15 days after CFC, unlike vehicle-treated rats, indicating that A_2A_R activation decreased fear generalization. These results seem at odds with our previous studies [19], where A_2A_R blockade before CFC decreased fear acquisition and memory [18, 19]. However, it is important to note that the engagement of A_2A_R throughout fear memory processing might differ. Indeed, CFC alters A_2A_R density in different regions of the fear circuitry, including hippocampus, basolateral amygdala, and ventral striatum [19]. Also, A_2A_R deletion from forebrain or from striatum has opposite consequences for fear acquisition and memory [18], but none of these previous studies investigated the role of A_2A_R in fear consolidation and generalization. Taken together, the data suggest that A_2A_R impact on fear is dependent on brain region and phase of fear memory processing and therefore A_2A_R may be manipulated at different time points and in opposite manners to control fear memories. Importantly, the present findings do not allow clarifying if the impact of A_2A_R on fear memory consolidation might be memory-strength dependent.

Newly acquired memories go through a gradual process of consolidation to become long-lasting [45]. Disturbances of this process may impair memory retrieval and/or specificity/accuracy of fear memories [30, 46–48]. CFC consolidation is particularly dependent on LTP in DH and LA [3, 4, 8]. Since A_2A_R control LTP in DH [29] and in LA [19] and affect fear memory [18, 19], we investigated if A_2A_R blockade immediately after CFC altered LTP in DH and/or LA. It is important to keep in mind that alterations of synaptic plasticity can result either from the acquisition and/or the consolidation of fear memory [3, 4, 8]; however, since SCH58261 was only applied after CFC, the effects of SCH58261 necessarily report the impact of A_2A_R on synaptic plasticity processes related to consolidation rather than on synaptic plasticity processes related to fear memory acquisition. Surprisingly, we observed that SCH58261 increased LTP magnitude both in DH and LA in CFC animals compared with rats injected with vehicle after CFC. This was only shown for conditions inducing robust LTP and it remains to be established if A_2A_R might affect the threshold of synaptic plasticity. The observed impact of SCH58261 on hippocampal and amygdala LTP may explain its effects on fear generalization since enhancement of DH-LTP is associated with enhancement of fear consolidation [49, 50] and increased LA-LTP during fear consolidation decreases fear memory accuracy leading to fear generalization [26, 51]. Again, these results seem at odds with our previous studies showing that A_2A_R blockade decreases both DH-LTP [25] and LA-LTP [19] and that A_2A_R blockade in both brain regions is associated with decreased fear learning and memory [18, 19]. They also seem at odds with the increased excitability of BLA principal neurons induced by A_2A_R activation [52] and the link between activation of the cAMP-PKA pathway (the canonical pathway triggered by A_2A_R) and increased LA neuronal excitability and fear generalization [26]. However, as previously mentioned, it is critical to consider that fear conditioning alters A_2A_R density in different brain regions of the fear circuitry [19]. Increased A_2A_R density is associated with a shift of function of A_2A_R, so that A_2A_R blockade decreases LTP in physiological conditions and increases LTP in pathological conditions associated with increased A_2A_R density [11, 16, 53–56]. For example, in different animal models of Alzheimer’s disease, A_2A_R are upregulated and A_2A_R blockade is associated with an increase (recovery) of hippocampal LTP and amelioration of memory deficits [54, 55], whereas A_2A_R blockade in control animals decreases LTP magnitude without affecting memory performance [54, 55]. A similar increase in A_2A_R density was observed at hippocampal and amygdala synapses after fear conditioning [19]. Thus, CFC-induced alterations in A_2A_R synaptic density may explain the opposite effect of A_2A_R blockade on fear responses when it happens before *versus* after CFC, although this still needs to be proven.

We further clarified the eventual involvement of adenosine A_1_ receptors (A_1_R) in the ability of A_2A_R to control fear generalization, since adenosine modulation of different brain functions and circuits involves a coordinated action of A_1_R and A_2A_R [34, 37–39]. We first observed that CFC decreased the potency of A_1_R activation to inhibit excitatory transmission in DH, but this did not alter the ability of A_2A_R to control CFC-induced alteration of DH-LTP. The involvement of A_1_R in A_2A_R-mediated effects on LA-LTP was less clear: although A_1_R function in LA was unaltered upon CFC, the A_1_R antagonist altered the effects of the A_2A_R antagonist on LTP both in control conditions and after CFC. This probably results from the peculiar pharmacology of adenosine receptors [41] and/or different circuit-mediated A_1_R/A_2A_R interactions in the amygdala [38], which still remain to be clarified. But overall, the present findings indicate that A_2A_R function is individually responsible for correcting aberrant plasticity, as occurs in DH after CFC, but might result from an interaction with A_1_R when synaptic plasticity is not overtly modified, as occurs in LA after CHC.

Altogether, these results show that targeting adenosine A_2A_R during fear consolidation can delay or accelerate fear generalization. This seems at least partially due to the control of LTP mechanisms occurring early on during memory consolidation at DH excitatory synapses. Based on our findings, it is proposed that A_2A_R agonists may be considered as a strategy to limit fear overgeneralization in trauma patients and to control symptoms in fear-related disorders, although it still remains to be defined how A_2A_R might control processes of fear extinction. Most importantly, the present findings may shed a new light on the overall impact of caffeine intake, the most widely consumed psychoactive drug [57], which selectively acts through the antagonism of adenosine receptors in non-toxic doses [57, 58]: in fact, caffeine may have opposite effects prophylactically decreasing fear acquisition and later therapeutically facilitating fear consolidation. This would prompt a recommendation to limit the intake of caffeinated coffee after emotionally traumatic events.

### Supplementary information

Legends to Supplementary files

Supplementary Fig. 1

Supplementary Fig. 2

## Data Availability

The data that support the findings of this study are available from the corresponding author upon reasonable request.
