# Supplementary material for: Adenosine A2A receptors control generalization of contextual fear in rats
Source: Transl Psychiatry. 2023 Oct 12;13:316. doi: 10.1038/s41398-023-02613-0 (PMC10570294; doi:10.1038/s41398-023-02613-0)
Supplement: Supplementary file 1 — Legends to Supplementary files [file 41398_2023_2613_MOESM1_ESM.docx]

**Supplementary Fig. 1 The blockade of A_2A_R immediately after context fear conditioning disrupts fear memory accuracy.** (A), (D) and (G) Scheme of the experimental design. Rats received vehicle or SCH58261 (0.1 mg/kg, i.p.) immediately after context fear conditioning (CFC). Individual values and mean ± SEM (n=8-11) of the percentage of time spent freezing in the context A (paired with foot-shocks) or in the context B (unpaired) first at days 1 and 2 (data not shown) and then at days 14 and 15 (data depicted in panels B and C). (B) The systemic administration of SCH58261 immediately after CFC enhanced fear behaviour at both contexts A and B, 14 and 15 days after CFC, respectively. (C) Discrimination index at 14 and 15 days after CFC. Both groups of animals do not discriminate between contexts A and B when probed for remote fear memory. (E) The effect of SCH58261 on the generalization of fear behaviour seems independent on the order of context presentation after CFC, although that the potentiating effect of SCH58261 on fear memory strength (in the paired context) seems absent when the paired context is tested second (confront with data presented in Figure 1). (F) Discrimination index at 1 and 2 days after CFC. Control (vehicle-treated) rats still discriminated between contexts A and B when presented to these contexts in an inverse order after CFC. However, SCH58261-treated rats were still unable to discriminate between contexts when probed for recent fear memory. (H) Animals subjected to immediate foot-shocks froze equally in contexts A and B, 1 and 2 days after the foot-shocks, respectively, regardless of treatment with SCH58261. (I) Discrimination index at 1 and 2 days after immediate foot-shocks. Both vehicle-treated (control) and SCH58261-treated animals were unable to discriminate between contexts A and B, 1 and 2 days, respectively, after receiving immediate foot-shocks in context A. (B) and (E) *p<0.05 in relation to the group treated with vehicle (one-way ANOVA followed by a Dunnett’s post-hoc test); (F) #p<0.05, one sample *t*-test when compared with the hypothetical value of 0.5 (no discrimination between contexts A and B).

**Supplementary Fig. 2 Fear conditioning triggers reproducible freezing behaviour.** Individual values and mean ± SEM (n=7-11) of the percentage of time spent in freezing behaviour of rats during 20 s starting 10 s after the last shock of low intensity (A, one shock at 0.3 mA), intermediate intensity (B, 3 shocks at 0.7 mA) and high intensity (C, 3 shocks at 1.2 mA), before administration of either SCH58261 (A, B; data after drug treatment displayed in Fig.1E and Fig.1B, respectively) or CGS21680 (C; data after drug treatment displayed in Fig.1B).
