# Supplementary figures and images for: Adenosine A2A receptors control generalization of contextual fear in rats
Source: Transl Psychiatry. 2023 Oct 12;13:316. doi: 10.1038/s41398-023-02613-0 (PMC10570294; doi:10.1038/s41398-023-02613-0)

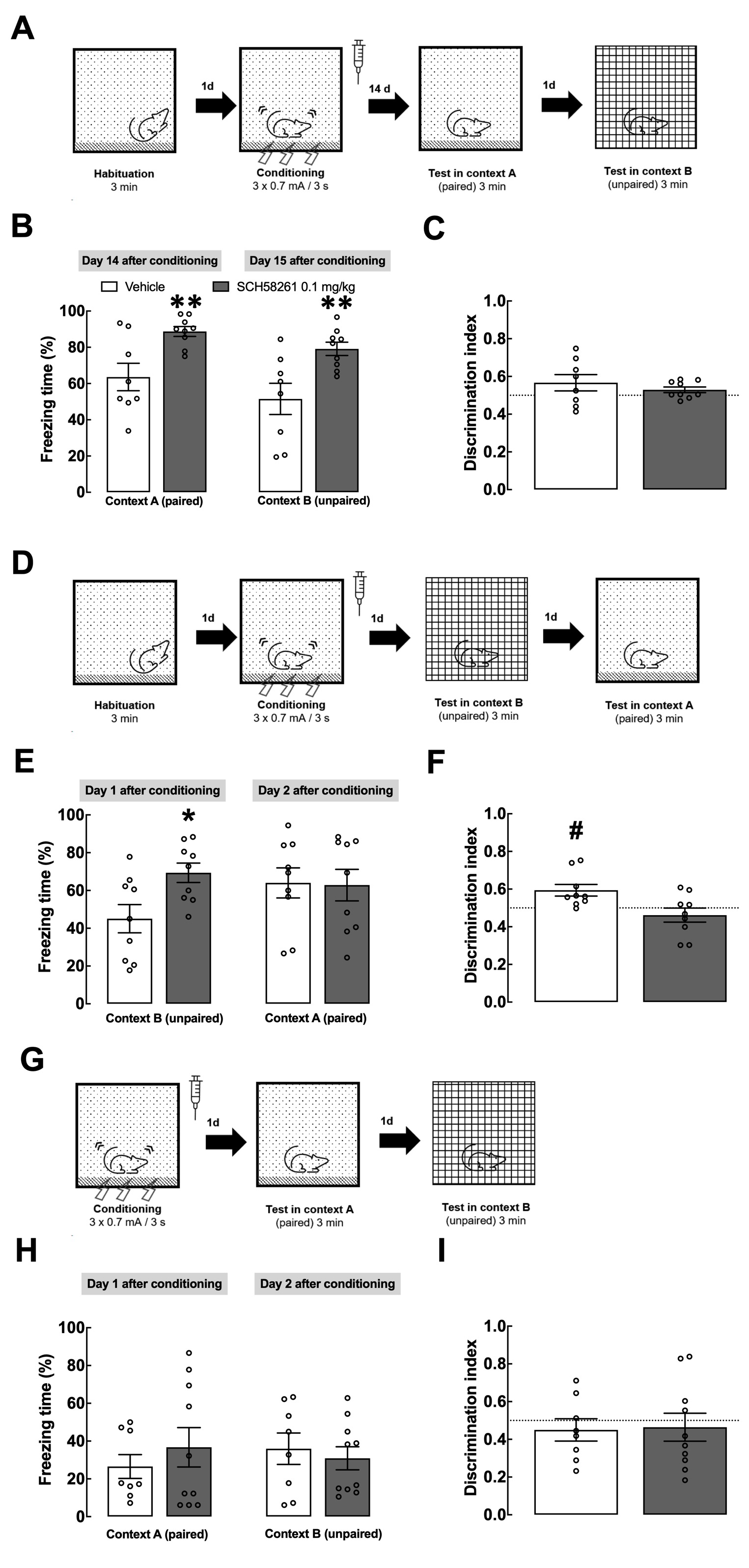

Supplement: Supplementary file 2 — Supplementary Fig. 1 [file 41398_2023_2613_MOESM2_ESM.jpg]

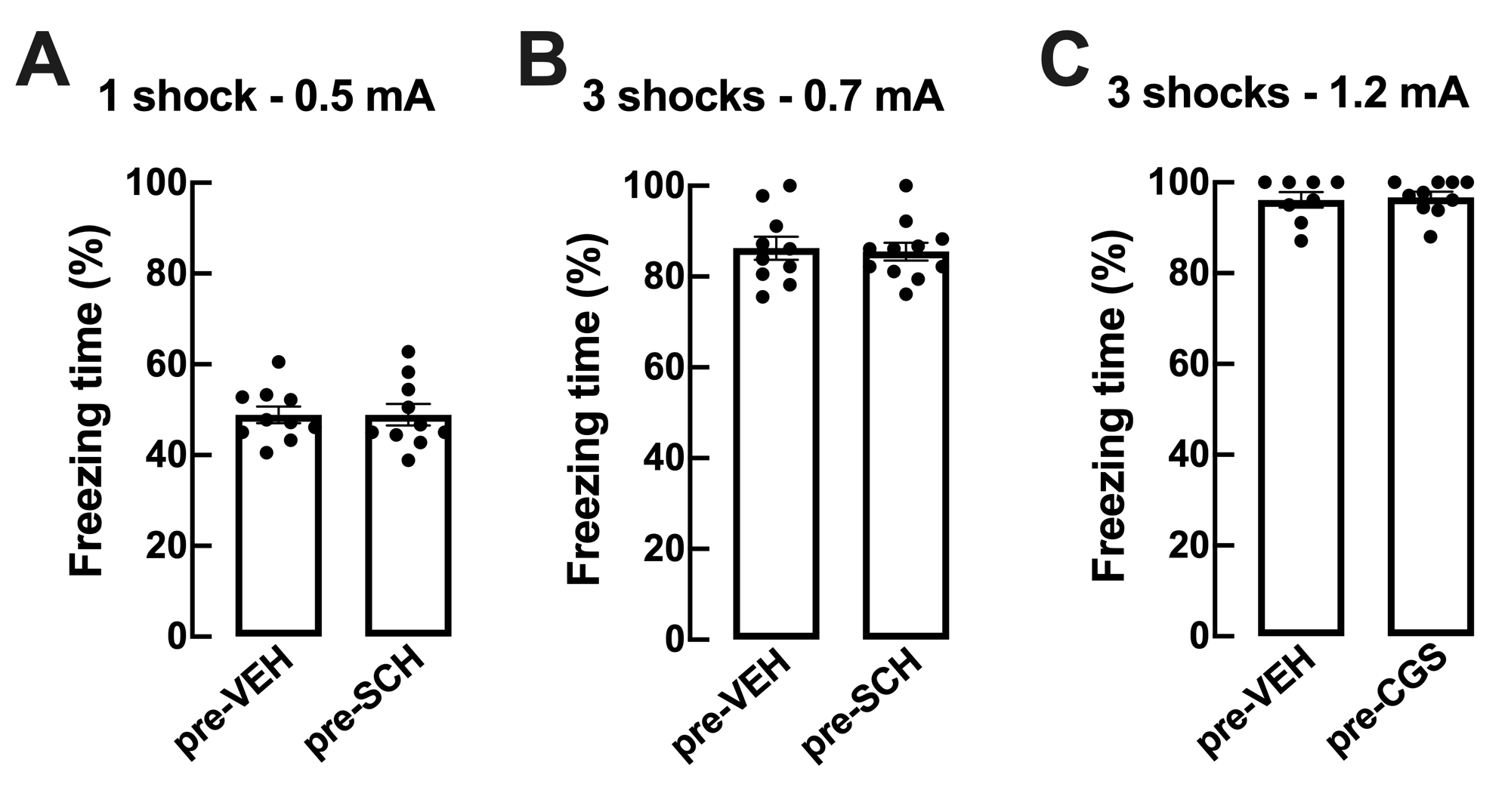

Supplement: Supplementary file 3 — Supplementary Fig. 2 [file 41398_2023_2613_MOESM3_ESM.tif]
